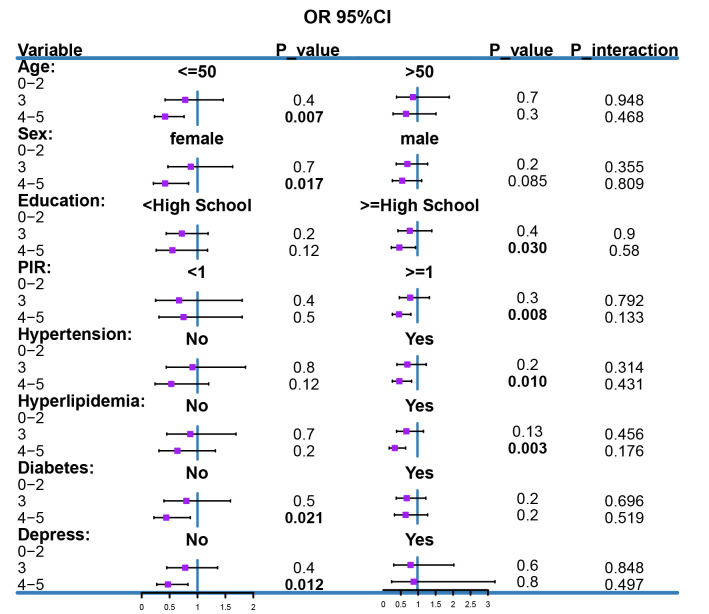# Association between healthy lifestyle factors and risk of chronic diarrhea: A cross-sectional study using NHANES 2007 to 2010 data

**DOI:** 10.1097/MD.0000000000049045

**Published:** 2026-05-29

**Authors:** JiRu Yuan, Xiong Chen, JieYun Wang, JunJie Luo, XueBin Shi, JunJiu Li

**Affiliations:** a Department of General Surgery, Dongguan Tungwah Hospital, Dongguan, Guangdong, China.

**Keywords:** chronic diarrhea, healthy lifestyle, healthy eating index, irritable bowel syndrome, NHANES, physical activity

## Abstract

This study aimed to investigate the association between a healthy lifestyle and the risk of chronic diarrhea. A total of 2921 eligible participants from the 2007 to 2010 National Health and Nutrition Examination Survey cycles were included in our study. We collected information on lifestyle factors such as diet, physical activity, sleep, smoking, and alcohol consumption, and defined chronic diarrhea as self-reported usual stool type corresponding to types 6 or 7 on the Bristol Stool Form Scale. The primary analysis used survey-weighted logistic regression to assess the association between healthy lifestyle behaviors and the risk of chronic diarrhea, and sensitivity analyses were performed to confirm the robustness of the findings. After adjusting for covariates, survey-weighted logistic regression showed that compared with the low lifestyle score group (0–2), the high lifestyle score group (4–5) was associated with a lower risk of chronic diarrhea (odds ratio [OR] = 0.49, 95% confidence interval [CI] = 0.29–0.82). When the high lifestyle score group was used as the reference, both the low-score group (OR = 2.06, 95% CI = 1.22–3.47) and the middle-score group (score of 3) (OR = 1.57, 95% CI = 1.03–2.39) were associated with a higher risk of chronic diarrhea. However, in the analysis of individual lifestyle factors after covariate adjustment, only the inverse association between diet and chronic diarrhea remained significant (OR = 0.58, 95% CI = 0.35–0.99). In addition, the interaction analysis did not identify any significant interactions among the covariates. A greater number of low-risk lifestyle factors may be associated with a lower risk of chronic diarrhea. Improving lifestyle behaviors holds substantial potential as a primary prevention strategy for chronic diarrhea, particularly among populations in economically underdeveloped regions.

## 1. Introduction

Diarrhea is a common gastrointestinal disease worldwide and often occurs secondary to bacterial or viral infections, posing a significant threat to human health.^[1]^ Global mortality from diarrhea remains high, especially in less developed regions such as Africa.^[2]^ Because sanitary conditions vary widely across countries and within populations, the incidence and mortality rates of diarrhea differ substantially.^[3]^ Targeted interventions can effectively reduce diarrhea-related deaths, but preventive and healthcare resources are unevenly distributed, and vulnerable groups in some regions may lack access even to safe drinking water and basic sanitation facilities.^[4,5]^ In addition, individual differences contribute to variations in clinical outcomes and recurrence rates.^[6]^ Given the widespread prevalence of diarrhea and its substantial impact on human health, developing effective and practical prevention strategies is particularly important, especially in low-income regions.

Adjusting modifiable lifestyle behaviors is a low-cost, practical, and effective approach to preventing and managing certain diseases. The influence of lifestyle factors on human health has been widely studied.^[7–9]^ Among these, the relationships among lifestyle, diet, and bowel habits have received considerable attention. A study based on the National Health and Nutrition Examination Survey (NHANES) database suggested that higher levels of healthy lifestyle behaviors may effectively reduce the incidence of diarrhea.^[10]^ Liu et al reported that dietary patterns can help maintain intestinal function through modulation of the gut microbiota, thereby reducing the risk of diarrhea.^[11]^ Given the strong association between gastrointestinal disorders and neuropsychiatric conditions, the underlying mechanisms are often attributed to the microbiota-gut-brain axis.^[12]^ The gut microbiota can influence the brain-gut axis through central nervous system pathways, enhancing synaptic transmission of serotonin, norepinephrine, and dopamine, thereby alleviating negative emotional states such as anxiety and depression.^[13]^ Animal studies have indicated that fatigue and high-fat diets may disrupt the gut microbiota, accompanied by intestinal inflammation and mucosal injury, ultimately leading to diarrhea.^[14]^ Smoking and heavy alcohol consumption have also been recognized as risk factors for gastrointestinal diseases.^[15]^ In contrast, physical activity (PA) has been shown to increase the diversity of beneficial gut bacteria and can alleviate irritable bowel syndrome (IBS) symptoms through the gut-brain axis, thereby reducing abdominal discomfort.^[16]^

The potential mechanisms linking lifestyle factors with gut health and the risk of diarrhea are multifaceted. A healthy diet rich in fiber and fermented foods promotes a diverse and beneficial gut microbiota, which is essential for maintaining intestinal barrier integrity and regulating immune function.^[17]^ Regular PA has been shown to reduce systemic and intestinal inflammation and may improve gut motility and visceral sensitivity through the gut-brain axis.^[18]^ In contrast, sleep deprivation and psychological stress can disrupt the composition of the gut microbiota, increase intestinal permeability, and exacerbate symptoms of diarrhea-predominant IBS via neuroendocrine pathways.^[19,20]^ Additionally, smoking and excessive alcohol consumption can damage the gastrointestinal mucosa and alter the gut microbiome, potentially increasing the risk of chronic diarrhea.^[15]^

Although a substantial body of research has examined the effects of lifestyle factors on gastrointestinal health or diarrhea,^[10,11,14–16]^ there remains a significant knowledge gap regarding the combined impact of multiple modifiable lifestyle factors (particularly key health-related behaviors such as diet, PA, sleep, smoking, and alcohol consumption) on the risk of chronic diarrhea in the general population. Moreover, most existing evidence is derived from studies focusing on IBS or specific patient groups, while large-scale, nationally representative data addressing overall lifestyle patterns and chronic diarrhea are scarce. This study aims to fill this gap by examining the association between a composite healthy lifestyle score incorporating these 5 key factors and the risk of chronic diarrhea. By evaluating both individual and combined effects, we seek to provide more comprehensive and practical evidence to inform lifestyle-based primary prevention strategies for chronic diarrhea, which is especially important in resource-limited settings.

This study utilized data from NHANES, a large, continuously updated, nationally representative survey that employs a complex, multistage, stratified sampling design. We selected participants from the 2007 to 2010 cycles to obtain information on healthy lifestyle factors and chronic diarrhea, and used logistic regression to examine their associations. This study may provide useful insights for the prevention and management of chronic diarrhea. In particular, for underdeveloped regions, such lifestyle-based strategies represent simple, low-cost, and feasible preventive measures that do not depend on economic or sanitary conditions.

## 2. Methods

### 2.1. Study population

A total of 20,686 participants from the 2007 to 2010 NHANES cycles were initially considered for our study. The inclusion and exclusion criteria were as follows: participants with missing data on diet, PA, sleep, smoking, or alcohol use were excluded (N = 10,999); participants with missing information on stool type were excluded (N = 687); participants with incomplete covariate data, including age, sex, race, body mass index (BMI), education level, poverty-to-income ratio (PIR), marital status, hypertension, diabetes, hyperlipidemia, and depression, were excluded (N = 5764); participants with constipation were excluded (N = 224); participants diagnosed with colorectal cancer were excluded (N = 39); pregnant women were excluded (N = 11); participants with ulcerative colitis were excluded (N = 15); participants with Crohn disease were excluded (N = 1); and participants who used laxatives were excluded (N = 25).

**Figure 1.**
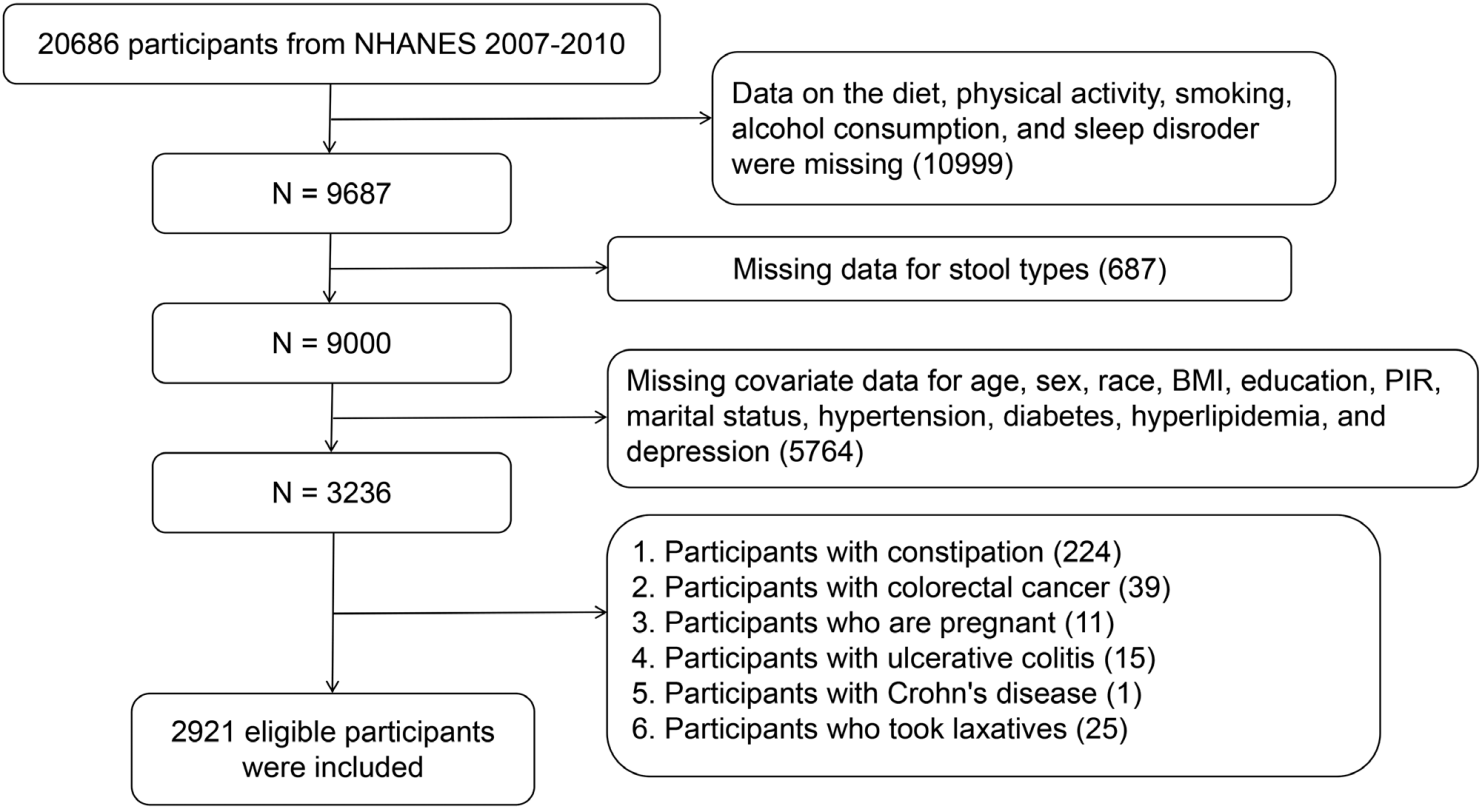
Flowchart for selecting eligible participant. BMI = body mass index, N = number of participants, PIR = poverty-to-income ratio.

After excluding participants with missing data or ineligible conditions according to the above criteria, a total of 2921 eligible participants were included in the final analysis. To improve the clarity and transparency of the participant selection process, the detailed screening procedure is presented in Figure 1.

### 2.2. Assessment of healthy lifestyle

The healthy lifestyle score was constructed based on prior large-scale epidemiological studies that have extensively examined the associations between lifestyle factors and the risk of chronic diseases.^[21–23]^ Drawing on this body of evidence, we included 5 modifiable lifestyle factors: diet, PA, sleep, smoking, and alcohol consumption.^[7,24–26]^ Dietary quality was assessed using the 2015 Healthy Eating Index (HEI),^[27]^ and the scoring criteria for dietary components are provided in Table S1. HEI scores were divided into quintiles, and scores above the third quintile were considered a low-risk factor. PA levels were obtained from the Global Physical Activity Questionnaire.^[28]^ Using the formula “(2 × vigorous-intensity activity + moderate-intensity activity) × duration per session,” we calculated each participant’s total weekly PA time across work and recreational activities. Weekly PA duration of < 3.5 hours was classified as a high-risk factor, with this threshold determined based on PA guidelines and prior epidemiological studies.^[28–30]^ Sleep disorders were identified based on responses to the question, “Has a doctor ever told you that you have a sleep disorder?” Individuals who answered “yes” were classified as having a sleep disorder.^[31]^ “Never smokers” were classified as a low-risk group, consistent with common public health definitions.^[25]^ Excessive alcohol consumption was considered a high-risk factor, defined as daily alcohol intake > 30 g for men and > 15 g for women. This definition is based on United States guidelines for moderate alcohol consumption and is consistent with those used in previous studies examining lifestyle factors and health outcomes.^[32,33]^

We treated these 5 lifestyle factors as independent components with equal weighting. Each low-risk lifestyle factor contributed 1 point to the healthy lifestyle score, whereas high-risk factors were assigned zero points. As a result, participants received a total healthy lifestyle score ranging from 0 to 5, with higher scores indicating a healthier lifestyle. Due to the limited number of participants with scores of 0, 1, or 5, treating the score as a continuous variable could introduce bias driven by extreme groups. Based on prior studies, we categorized the score into 3 groups: low (0–2), intermediate (3), and high (4–5), to minimize bias and enhance statistical power.^[7,24]^

### 2.3. Assessment of chronic diarrhea

The definition of chronic diarrhea was based on participants’ self-reported stool type. The NHANES gastrointestinal health data include the Bristol Stool Form Scale,^[34]^ in which participants report their usual stool type during the interview. Specifically, type 1 (separate hard lumps, like nuts) and type 2 (sausage-shaped but lumpy) were classified as chronic constipation. Types 3 (sausage-like with cracks on the surface), 4 (sausage or snake-like, smooth and soft), and 5 (soft blobs with clear-cut edges) were categorized as normal stool. Types 6 (fluffy pieces with ragged edges, mushy stool) and 7 (watery, no solid pieces) were classified as chronic diarrhea. Participants with chronic constipation or missing Bristol Stool Form Scale data were excluded. In addition, because IBS-related diarrhea is very common in the population, it was necessary to include these individuals in the analysis of chronic diarrhea; therefore, IBS-related diarrhea was not excluded. Eligible participants were ultimately classified into 2 groups: chronic diarrhea and normal stool.

### 2.4. Covariates

Based on previous studies, to minimize potential confounding and improve the accuracy of our findings, we included the following covariates.^[11,35]^ Age, sex, and race (Mexican American, other Hispanic, non-Hispanic White, non-Hispanic Black, and other) were incorporated, along with education level (< high school, ≥ high school). BMI was categorized as overweight when BMI > 25 and normal when ≤ 25. The PIR was classified as PIR ≥ 1 or PIR < 1. Marital status was grouped as living alone, married or living with partner. In addition, hypertension, diabetes, hyperlipidemia, and depression were included.

Hypertension was defined as a prior physician diagnosis or a mean systolic blood pressure ≥ 140 mm Hg or mean diastolic blood pressure ≥ 90 mm Hg across 3 measurements. Diabetes was defined as HbA1c ≥ 6.5%, fasting glucose ≥ 126 mg/dL, a previous diagnosis, or current insulin use. Hyperlipidemia was identified when participants answered “yes” to the question, “Have you ever been told by a doctor or other health professional that your blood cholesterol level was high?”

Depression was assessed using the Patient Health Questionnaire.^[36]^ Based on responses to 9 items covering depressive symptoms over the past 2 weeks (including mood, sleep, psychomotor changes, appetite, and suicidal ideation) each item was scored from 0 (not at all) to 3 (nearly every day), yielding a total score of 0 to 27. A total score ≥ 10 was classified as depression.

### 2.5. Statistical analysis

This study adopted the complex stratified sampling design of the NHANES database and incorporated sampling weights, stratification variables, and primary sampling unit variables to ensure national representativeness and to obtain robust standard errors. We treated chronic diarrhea as a binary outcome variable and used survey-weighted logistic regression to evaluate the associations of individual and overall healthy lifestyle factors with chronic diarrhea. Restricted cubic splines (RCS) analyses and interaction analyses were also performed under survey-weighted conditions. We used variance inflation factors based on the weighted design matrix to evaluate multicollinearity. The results showed that all independent variables had variance inflation factors values below 5, indicating that there was no serious multicollinearity after accounting for the survey design. To assess the robustness of our findings, we conducted model-based sensitivity analyses. Three nested models were constructed: model 1: unadjusted; model 2: adjusted for age, sex, race, and education; and model 3: which further adjusted for BMI, PIR, marital status, hypertension, diabetes, hyperlipidemia, and depression. In addition, we examined the robustness of the results by modifying the grouping definition of the healthy lifestyle score and comparing the results from 2 different classification approaches. This allowed us to observe potential differences and further strengthen the credibility of our findings.

All statistical analyses were conducted using R version 4.5.0 (R Foundation for Statistical Computing). Two-sided tests were used to determine statistical significance, with a *P* value < .05 considered statistically significant.

## 3. Results

### 3,1. Basic characteristics of the study population

As shown in Table 1, the 2921 participants were categorized into 2 groups based on the presence of chronic diarrhea. Participants with chronic diarrhea were more often female and had lower educational attainment and PIR levels, along with higher prevalences of hypertension, diabetes, and depression. Significant differences were also observed between groups in smoking status, sleep disorders, HEI, and PA. Individuals with chronic diarrhea had higher rates of smoking and sleep disorders, as well as lower HEI scores and shorter PA durations.

**Table 1:** Baseline characteristics of participants stratified by chronic diarrhea status.

	Diarrhea		
Characteristic	No, N = 2642 (91%)^*^	Yes, N = 279 (8.8%)^*^	*P* Value^†^
Age (yrs)	54 (43, 65)	52 (44, 66)	.4
Gender			**.032**
Female	1313 (51%)	156 (59%)	
Male	1329 (49%)	123 (41%)	
Race			.2
Mexican American	369 (5.3%)	42 (5.5%)	
Other Hispanic	241 (3.3%)	28 (4.1%)	
Non-Hispanic White	1441 (77%)	144 (75%)	
Non-Hispanic Black	502 (9.8%)	56 (13%)	
Other/multiracial	89 (4.9%)	9 (2.8%)	
Education			**< .001**
< High School	645 (16%)	108 (27%)	
≥ High School	1996 (84%)	171 (73%)	
BMI group			.7
Normal	583 (25%)	47 (23%)	
Overweight	2059 (75%)	232 (77%)	
PIR			**0.007**
< 1	391 (9.6%)	72 (16%)	
≥ 1	2251 (90%)	207 (84%)	
Marital			0.6
Living alone	942 (31%)	102 (32%)	
Married or living with partner	1699 (69%)	176 (68%)	
Hypertension			**.006**
No	1146 (51%)	88 (42%)	
Yes	1496 (49%)	191 (58%)	
Diabetes			**.041**
No	1727 (73%)	152 (65%)	
Yes	915 (27%)	127 (35%)	
Hyperlipidemia			.9
No	1348 (53%)	129 (54%)	
Yes	1294 (47%)	150 (46%)	
Depression			**< .001**
No	2438 (94%)	224 (83%)	
Yes	204 (6.3%)	55 (17%)	
Smoking status			**.044**
Current smoker	416 (15%)	56 (23%)	
Former smoker	824 (30%)	93 (32%)	
Never smoker	1402 (54%)	130 (45%)	
Excessive alcohol consumption			.5
No	2382 (88%)	257 (91%)	
Yes	260 (12%)	22 (9.3%)	
Sleep disorder			**.042**
No	2387 (91%)	238 (86%)	
Yes	255 (9.0%)	41 (14%)	
Healthy eating index (0–100)	52 (44, 61)	50 (42, 59)	**.047**
Physical activity (hr/wk)	5 (0, 15)	2 (0, 10)	**.022**
Healthy life score			**.002**
0–2	755 (26%)	110 (39%)	
3	1051 (39%)	106 (40%)	
4–5	836 (35%)	63 (21%)	

BMI = body mass index, IQR = interquartile ratio, n/N = number of participants, PIR = poverty-to-income ratio.

* Median (IQR); n (unweighted) (%).

† Wilcoxon rank-sum test for complex survey samples; chi-squared test with Rao & Scott second-order correction.

Given the substantial variation in the distribution of healthy lifestyle scores across participants, we classified the scores into 3 categories: low (0–2), intermediate (3), and high (4–5). Notably, the distribution of healthy lifestyle scores differed significantly between the 2 groups. Participants with chronic diarrhea had a higher proportion of low scores and a lower proportion of high scores compared with those without chronic diarrhea.

### 3.2. Association between healthy lifestyle and risk of chronic diarrhea

Survey-weighted logistic regression was used to evaluate the association between a healthy lifestyle and the risk of chronic diarrhea. When using the 0 to 2 group as the reference, individuals with a healthy lifestyle score of 4 to 5 had a lower risk of chronic diarrhea (odds ratio [OR] = 0.49, 95% confidence interval [CI] = 0.29–0.82), even after adjusting for covariates (Table 2). When using the 4 to 5 group as the reference, the adjusted results showed that participants with a score of 3 (OR = 1.57, 95% CI = 1.03–2.39) and those with a score of 0 to 2 (OR = 2.06, 95% CI = 1.22–3.47) had a higher risk of chronic diarrhea (Table 3).

**Table 2:** Association between healthy lifestyle and chronic diarrhea risk (reference: score 0–2).

	Model 1			Model 2			Model 3		
Characteristic	OR^1^	95% CI^1^	*P* value	OR^1^	95% CI^1^	*P* value	OR	95% CI	*P* value
Healthy_lifestyle									
0–2		-		-	-		-	-	
3	0.69	0.43, 1.10	.12	0.72	0.45, 1.14	.2	0.76	0.46, 1.27	.3
4–5	0.41	0.26, 0.65	**< .001**	0.45	0.29, 0.71	**.001**	0.49	0.29, 0.82	**.010**

Model 1: Unadjusted; Model 2: Adjusted for age, sex, race, and education; Model 3: Adjusted for age, sex, race, education, BMI, PIR, marital status, hypertension, diabetes, hyperlipidemia, and depression.

BMI = body mass index, CI = confidence interval, OR = odds ratio, PIR = poverty-to-income ratio.

*P* < .05 is highlighted in bold.

**Table 3:** Association between healthy lifestyle and chronic diarrhea risk (reference: score 4–5).

	Model 1			Model 2			Model 3		
Characteristic	OR	95% CI	*P* value	OR	95% CI	*P* value	OR	95% CI	*P* value
Healthy_lifestyle									
4–5	-	-		-	-		-	-	
3	1.70	1.18, 2.45	**.006**	1.58	1.10, 2.27	**.016**	1.57	1.03, 2.39	**.036**
0–2	2.45	1.55, 3.89	**< .001**	2.20	1.41, 3.45	**.001**	2.06	1.22, 3.47	**.010**

Model 1: Unadjusted; Model 2: Adjusted for age, sex, race, and education; Model 3: Adjusted for age, sex, race, education, BMI, PIR, marital status, hypertension, diabetes, hyperlipidemia, and depression.

BMI = body mass index, CI = confidence interval, OR = odds ratio, PIR = poverty-to-income ratio.*P* < .05 is highlighted in bold.

### 3.3. Relationship between individual healthy lifestyle factors and the risk of chronic diarrhea

To examine the individual effects of the 5 lifestyle factors on the risk of chronic diarrhea, we conducted survey-weighted logistic regression analyses for diet, PA, sleep, smoking, and alcohol intake. The unadjusted results showed that low-risk levels of diet, PA, and sleep were associated with a lower risk of chronic diarrhea. However, after fully adjusting for all included covariates, only the association between diet and chronic diarrhea remained statistically significant (OR = 0.58, 95% CI = 0.35–0.99), whereas the associations for PA and sleep were no longer significant (Table 4).

**Table 4:** Associations between individual healthy lifestyle factors and chronic diarrhea risk.

	Model 1			Model 2			Model 3		
Characteristic	OR	95% CI	*P* value	OR	95% CI	*P* value	OR	95% CI	*P* value
HEI									
high_risk	-	-		-	-		-	-	
low_risk	0.59	0.38, 0.92	**.022**	0.58	0.35, 0.98	**.041**	0.58	0.35, 0.99	**.045**
PA									
high_risk	-	-		-	-		-	-	
low_risk	0.61	0.41, 0.89	**.013**	0.68	0.45, 1.02	.063	0.72	0.48, 1.10	.12
Sleep									
high_risk	-	-		-	-		-	-	
low_risk	0.61	0.38, 0.98	**.044**	0.61	0.38, 0.97	**.039**	0.75	0.48, 1.18	.2
Smoke									
high_risk	-	-		-	-		-	-	
low_risk	0.67	0.44, 1.02	.062	0.70	0.46, 1.05	.080	0.73	0.48, 1.10	.13
Drink									
high_risk	-	-		-	-		-	-	
low_risk	1.29	0.61, 2.74	.5	1.26	0.59, 2.65	.5	1.17	0.55, 2.48	.7

Model 1: Unadjusted; Model 2: Adjusted for age, sex, race, and education; Model 3: Adjusted for age, sex, race, education, BMI, PIR, marital status, hypertension, diabetes, hyperlipidemia, and depression.

BMI = body mass index, CI = confidence interval, HEI = healthy eating index, OR = odds ratio, PA = physical activity, PIR = poverty-to-income ratio.

*P* < .05 is highlighted in bold.

### 3.4. RCS regression analysis

**Figure 2.**
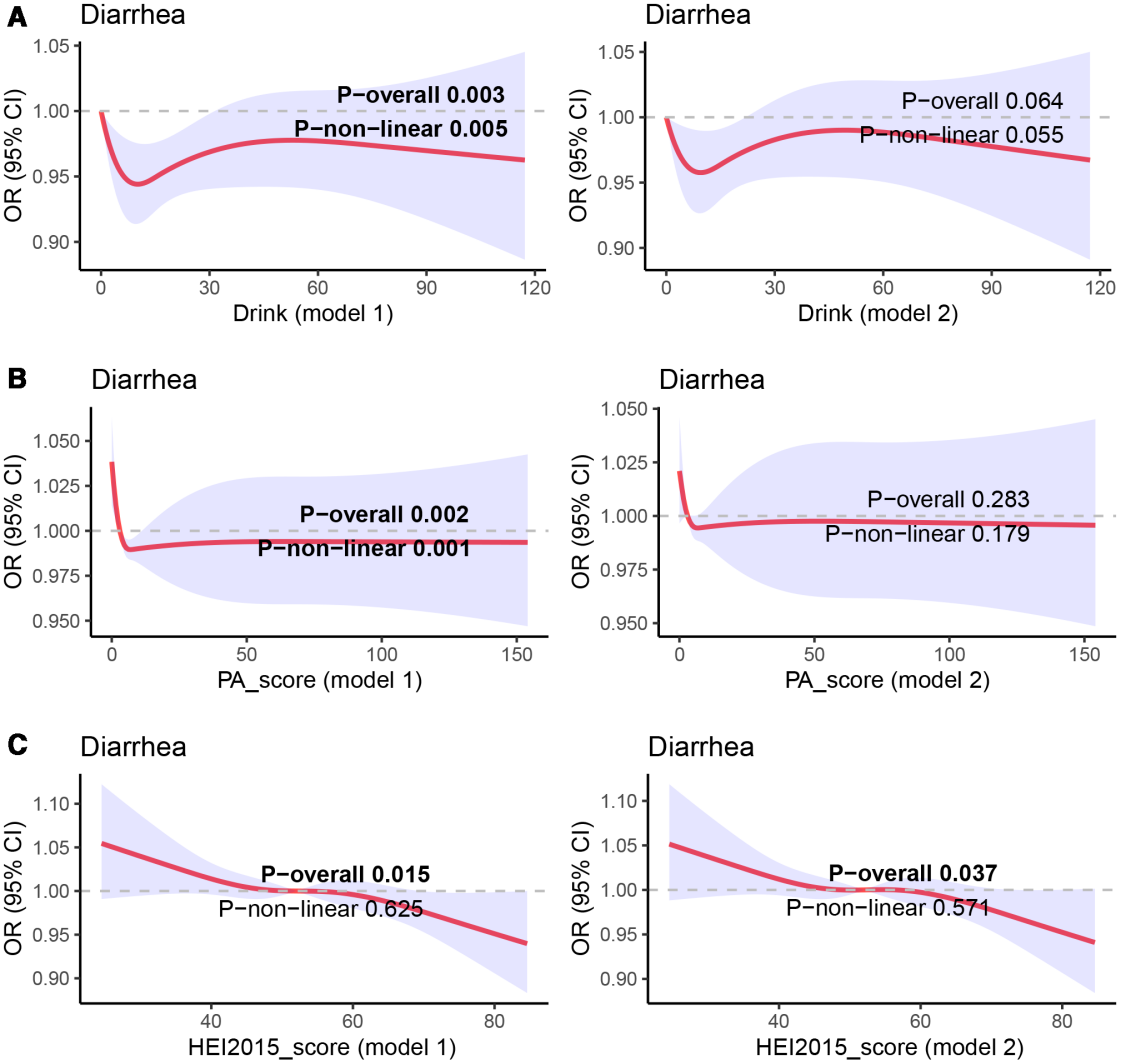
RCS regression analyses of alcohol intake, PA time, and HEI. (A) Alcohol intake and chronic diarrhea risk; (B) PA time and chronic diarrhea risk; (C) HEI and chronic diarrhea risk. The red curve represents the nonlinear fitted association, and the light red shaded area indicates the corresponding confidence range. Model 1: unadjusted; Model 2: adjusted for age, sex, race, BMI, education level, PIR, marital status, hypertension, diabetes, hyperlipidemia, and depression. Results with *P* < .05 are shown in bold. BMI = body mass index, CI = confidence interval, HEI = Healthy Eating Index, NHANES = National Health and Nutrition Examination Survey, OR = odds ratio, PA = physical activity, PIR = poverty-to-income ratio, RCS = restricted cubic splines.

To further examine the relationship between a healthy lifestyle and the risk of chronic diarrhea, we conducted survey-weighted RCS analyses to explore potential nonlinear associations of HEI, PA time, and alcohol intake with chronic diarrhea. The unadjusted RCS models showed significant linear associations for alcohol intake, PA time, and HEI with chronic diarrhea (*P* overall < .05). Even after adjustment for covariates, HEI remained significantly and negatively associated with chronic diarrhea risk (*P* overall for HEI = .037), with higher HEI scores corresponding to a progressively lower risk of chronic diarrhea (Fig. 2C). However, after adjustment, the RCS associations for PA and alcohol intake were no longer statistically significant (Fig. 2A and 2B).

### 3.5. Subgroup analyses and interaction tests

To explore whether the association between a healthy lifestyle and chronic diarrhea differed across populations, we conducted subgroup analyses and interaction tests. As shown in Figure S1, subgroup analyses demonstrated a consistent inverse association between the healthy lifestyle score and the risk of chronic diarrhea across all strata, including age (≤ 50 vs > 50 years), sex (men vs women), education level (< high school vs ≥ high school), PIR (< 1 vs ≥ 1), and the presence of hypertension, hyperlipidemia, diabetes, or depression. That is, higher scores were consistently associated with lower risk.

However, interaction tests indicated no statistically significant effect modification by age, sex, education level, PIR, hypertension, hyperlipidemia, diabetes, or depression (all *P* for interaction > .05). This suggests that although point estimates varied slightly across subgroups, these differences were not statistically significant. Overall, our analysis found no evidence that age, sex, socioeconomic status, or the included comorbidities meaningfully modified the association between a healthy lifestyle and the risk of chronic diarrhea.

### 3.6. Sensitivity analysis

To further verify the reliability and robustness of the results, we changed the grouping of the healthy lifestyle score from the original tertile classification (0–2 as the low group, 3 as the middle group, 4–5 as the high group) to a new tertile classification (0–1 as the low group, 2–3 as the middle group, 4–5 as the high group). Under the new grouping scheme, when the low group (0–1) was used as the reference, the high group (4–5) remained significantly associated with a lower risk of chronic diarrhea (OR = 0.43, 95% CI = 0.21–0.90) (Table S2), which was similar to the main analysis result (OR = 0.49, 95% CI = 0.29–0.82), with a difference of < 20%. In addition, when the high group (4–5) was used as the reference, similar results were observed. The middle group (2–3) (OR = 1.72, 95% CI = 1.16–2.56) and the low group (0–1) (OR = 2.31, 95% CI = 1.11–4.82) were both associated with a higher risk of chronic diarrhea (Table S3). These findings indicate that our results are not sensitive to the scoring classification method and demonstrate good robustness.

## 4. Discussion

Based on the correlation analyses using NHANES data, we found that healthier lifestyle behaviors appear to reduce the risk of chronic diarrhea. Sensitivity analyses using alternative model specifications further confirmed the robustness of our results. However, after adjusting for covariates in the individual lifestyle factor analyses, only the diet score remained significantly associated with chronic diarrhea risk. Many previous studies have demonstrated that unhealthy dietary patterns can trigger and promote the development of diarrhea, which is consistent with our findings.^[14,37,38]^ Dietary modification and restriction therapies have been widely used to manage gastrointestinal disorders such as IBS, diarrhea, bloating, and constipation.^[39,40]^ Examples include traditional dietary advice, the low fermentable oligosaccharides, disaccharides, monosaccharides, and polyols diet, and the gluten free diet, which have shown significant benefits in some populations.^[41,42]^ However, because of individual differences in physiology and variations in the gut microbiota, there is still no ideal nutritional regimen for patients with chronic diarrhea.^[42]^

In addition, IBS and functional gastrointestinal disorders that often lead to chronic diarrhea are now considered disorders of gut brain interaction and are closely related to the gut microbiota, intestinal barrier function, gastrointestinal motility disorders, intestinal immune regulation, and psychological factors.^[43,44]^ Some central neuromodulators are also used in the treatment of IBS.^[13]^ It has even been suggested that certain central nervous system disorders may be improved through dietary approaches that benefit the gut microbiota and the gut brain axis.^[45]^ *Bifidobacterium* and *Lactobacillus* are currently among the most commonly used probiotics. Studies have shown that probiotics can increase levels of butyrate, tryptophan, and N acetyltryptophan, thereby improving anxiety and depression and reducing amygdala activation.^[46]^ On the other hand, clinical and experimental studies have shown that psychological stress has significant effects on intestinal sensitivity, motility, secretion, and permeability. These effects are associated with mucosal immune activation and changes in the central nervous system, peripheral neurons, and the gut microbiota.^[47]^ Medications such as antidepressants, antipsychotics, and 5 hydroxytryptamine synthesis inhibitors also play important roles in the treatment of IBS.^[47]^ Therefore, chronic diarrhea should not be viewed simply as a gastrointestinal disorder but rather as a condition that requires integrated consideration of the nervous, endocrine, and digestive systems. Given the complexity of chronic diarrhea, it is necessary to include lifestyle factors such as sleep, PA, smoking, and alcohol consumption in the analysis, which provides a more accurate and comprehensive perspective for exploring their associations.

In this study, the prevalence of chronic diarrhea was 8.8%. Individuals diagnosed with chronic diarrhea were more likely to be female, have lower educational attainment and lower PIR, and had higher rates of hypertension, diabetes, and depression. The prevalence of chronic diarrhea and related abdominal discomfort appears to be higher among women and individuals in underdeveloped regions.^[3,4,48]^ This may be attributed to stronger gut-brain interactions and more pronounced psychosocial disturbances in women,^[49,50]^ as well as less developed preventive healthcare systems in economically disadvantaged areas.^[3]^ Therefore, greater attention to gastrointestinal health and targeted prevention strategies is warranted for women and populations in underdeveloped regions. Improving lifestyle behaviors may represent a cost-effective, practical, and scalable approach for the prevention of chronic diarrhea.

Using survey-weighted logistic regression, we found that participants with higher healthy lifestyle scores had a significantly lower risk of chronic diarrhea compared with those in the low-score group. To further verify this association, we reversed the reference group and used the high-score group as the baseline; the results showed that both the low- and middle-score groups had higher risks of chronic diarrhea. Sensitivity analyses yielded consistent findings, supporting the robustness of our results. These observations suggest that, when considering the combined effects of 5 low-risk lifestyle factors (diet, PA, sleep, smoking, and alcohol use) a healthier lifestyle overall could be associated with a reduced risk of chronic diarrhea. Previous studies have shown that higher life’s essential-8 levels are associated with a lower incidence of diarrhea.^[10]^ Another study using data from the United Kingdom Biobank reported that a greater number of healthy lifestyle behaviors was linked to a reduced risk of IBS.^[35]^ These findings are consistent with our results. Research on individual lifestyle factors and chronic diarrhea is also extensive. For example, consumption of cold drinks, high-fat foods, and other unhealthy dietary patterns can increase the risk of diarrhea, potentially through gut microbiota dysbiosis and impaired intestinal mucosal barrier function, often accompanied by intestinal inflammation.^[14,37,38,51,52]^ PA, as a non-pharmacologicalintervention, has been shown to enhance the diversity and abundance of beneficial gut bacteria, exert anti-inflammatory effects, and support intestinal barrier integrity.^[16]^ Additionally, PA has significant benefits for mental health and can alleviate symptoms such as diarrhea and bloating through the gut-brain axis.^[16]^ Bouchoucha et al reported that sleep disturbances are associated with functional gastrointestinal disorders, including diarrhea, bloating, and fecal incontinence.^[53]^ Smoking has been linked in multiple studies to increased intestinal inflammation and higher risk of IBS,^[15,54]^ while excessive alcohol consumption has been shown to cause damage across all segments of the gastrointestinal tract.^[55]^

To further evaluate the effects of the 5 lifestyle factors (diet, PA, sleep, smoking, and alcohol use) on chronic diarrhea, and to compare their overall and individual impacts, we conducted logistic regression analyses for each factor. Although HEI, PA, and sleep were all significantly and inversely associated with chronic diarrhea risk in the unadjusted models, only the association between HEI and chronic diarrhea remained significant after fully adjusting for covariates. Consistent results were observed in the RCS analyses, which showed a decreasing risk of chronic diarrhea with higher HEI scores. In addition, interaction tests did not identify any significant interaction effects. These findings suggest that the overall and individual effects of healthy lifestyle behaviors on chronic diarrhea risk may differ. In other words, the combined effects of healthy behaviors including diet, PA, sleep, smoking, and alcohol consumption may be more effective in reducing the risk of chronic diarrhea than adhering to any single lifestyle factor alone. It should be noted that this does not imply that factors such as PA, sleep, smoking, and alcohol consumption are unrelated to chronic diarrhea. Larger cohort studies and more in-depth mechanistic research are needed to further validate and clarify these observations.

The mechanisms through which lifestyle improvements may help prevent chronic diarrhea are diverse. Chronic diarrhea frequently occurs in patients with IBS, and given the high comorbidity between gastrointestinal disorders and neuropsychiatric conditions, increasing attention has been directed to the importance of the microbiota-gut-brain axis.^[12]^ Healthy dietary patterns and adequate PA exert significant regulatory effects on the gut microbiota and promote the growth of beneficial bacteria.^[42]^ Probiotics have been shown to prevent and improve digestive diseases by modulating the immune system.^[56]^ In addition, the gut microbiota can influence endocrine and neural functions. Central neuromodulators can enhance synaptic transmission of serotonin, norepinephrine, and dopamine, acting on receptors along the brain-gut axis to alleviate negative emotional states such as anxiety and depression.^[13,46]^ As a result, many researchers have suggested that the management of diarrhea should be integrated with the treatment of mental disorders. On the other hand, insufficient sleep and sleep disturbances increase the risk of anxiety and depression,^[57,58]^ which may in turn lead to gastrointestinal symptoms such as diarrhea. Moreover, the harmful effects of nicotine on the gastrointestinal tract have been widely documented,^[15]^ and alcohol can inhibit sodium and water absorption, leading to diarrhea in heavy drinkers.^[59]^ Therefore, maintaining a healthy diet and adequate PA, ensuring proper sleep, and limiting smoking and alcohol consumption may be effective strategies for preventing chronic diarrhea.

Our study used the NHANES database to conduct a large-scale cross-sectional analysis examining the impact of a healthy lifestyle on the risk of chronic diarrhea. Although our findings on lifestyle-based prevention and management strategies for chronic diarrhea are encouraging, it is important to acknowledge several key limitations of our study. First, the cross-sectional design does not allow causal inference. Second, the definition of chronic diarrhea was based on self-reported questionnaires, which may introduce recall bias. Third, although we adjusted for covariates as comprehensively as possible, residual confounding from certain unmeasured or unavailable factors (such as the lack of data on antibiotic use, dietary intolerance, thyroid disease, and other related gastrointestinal diseases) cannot be completely ruled out. Fourth, the measurement and dichotomization of lifestyle factors, while based on common standards, may not capture the full complexity of these behaviors and could introduce some degree of exposure misclassification. Finally, the study population consisted solely of United States participants, and research involving more diverse racial and ethnic groups is needed to determine the generalizability of our findings. These limitations suggest that our findings should be interpreted as preliminary associative evidence, and their causal nature and generalizability require further validation in future studies.

## Conclusion

In this cross-sectional study based on nationally representative data, adherence to a greater number of healthy lifestyle behaviors was associated with a lower risk of chronic diarrhea. These findings suggest that comprehensive lifestyle modification may serve as an effective strategy for the primary prevention of chronic diarrhea. Further prospective cohort studies and intervention trials are warranted to confirm these associations and clarify causality.

## Acknowledgments

We sincerely thank NHANES for providing all of the data.

## Author contributions

**Conceptualization:** JiRu Yuan, JunJiu Li.

**Data curation:** JiRu Yuan, JunJie Luo.

**Formal analysis:** Xiong Chen, JieYun Wang.

**Methodology:** Xiong Chen, JieYun Wang, XueBin Shi.

**Software:** Xiong Chen.

**Visualization:** Xiong Chen.

**Writing – original draft:** JiRu Yuan.

**Writing – review & editing:** Xiong Chen, JieYun Wang, JunJiu Li.



**Figure:**