# Supplementary material for: Association between healthy lifestyle factors and risk of chronic diarrhea: A cross-sectional study using NHANES 2007 to 2010 data
Source: Medicine (Baltimore). 2026 May 29;105(22):e49045. doi: 10.1097/MD.0000000000049045 (PMC13225563; doi:10.1097/MD.0000000000049045)
Supplement: Supplementary file 1 [file medi-105-e49045-s001.docx]

|  |  | **Healthy Eating Index-2015 (Applies to Ages 2 y and Older)** | |
| --- | --- | --- | --- |
| **Component** | **Maximum points** | **Standard for maximum score** | **Standard for minimum score of 0** |
| **Adequacy components** |  |  |  |
| Total Fruits^b^ | 5 | ≥0.8 cup eq/1,000 kcal | No Fruit |
| Whole Fruits^c^ | 5 | ≥0.4 cup eq/1,000 kcal | No Whole Fruit |
| Total Vegetables^d^ | 5 | ≥1.1 cup eq/1,000 kcal | No Vegetables |
| Greens and Beans^d^ | 5 | ≥0.2 cup eq/1,000 kcal | No Dark Green Vegetables or Legumes |
| Whole Grains | 10 | ≥1.5 oz eq/1,000 kcal | No Whole Grains |
| Dairye | 10 | ≥1.3 cup eq/1,000 kcal | No Dairy |
| Total Protein Foods^d^ | 5 | ≥2.5 oz eq/1,000 kcal | No Protein Foods |
| Seafood and Plant Proteins^f^ | 5 | ≥0.8 oz eq/1,000 kcal | No Seafood or Plant Proteins |
| Fatty Acids^g^ | 10 | (PUFAs^h^ + MUFAs^i^) / SFAs^j^ ≥2.5 | (PUFAs + MUFAs) / SFAs ≤1.2 |
| **Moderation components** |  |  |  |
| Refined Grains | 10 | ≤1.8 oz eq/1,000 kcal | ≥4.3 oz eq/1,000 kcal |
| Sodium | 10 | ≤1.1 g/1,000 kcal | ≥2.0 g/1,000 kcal |
| Added Sugars | 10 | ≤6.5% of energy | ≥26% of energy |
| Saturated Fats | 10 | ≤8% of energy | ≥16% of energy |
