# Supplementary material for: Association between healthy lifestyle factors and risk of chronic diarrhea: A cross-sectional study using NHANES 2007 to 2010 data
Source: Medicine (Baltimore). 2026 May 29;105(22):e49045. doi: 10.1097/MD.0000000000049045 (PMC13225563; doi:10.1097/MD.0000000000049045)
Supplement: Supplementary file 2 [file medi-105-e49045-s003.docx]

|  | model1 | | | model2 | | | model3 | | |
| --- | --- | --- | --- | --- | --- | --- | --- | --- | --- |
| **Characteristic** | **OR**^1^ | **95% CI**^1^ | **p-value** | **OR**^1^ | **95% CI**^1^ | **p-value** | **OR**^1^ | **95% CI**^1^ | **p-value** |
| **healthy_lifestyle** |  |  |  |  |  |  |  |  |  |
| *0-1* | — | — |  | — | — |  | — | — |  |
| *2-3* | 0.62 | 0.31, 1.25 | 0.2 | 0.64 | 0.32, 1.29 | 0.2 | 0.75 | 0.38, 1.45 | 0.4 |
| *4-5* | 0.32 | 0.15, 0.69 | **0.005** | 0.36 | 0.17, 0.76 | **0.009** | 0.43 | 0.21, 0.90 | **0.028** |
| ^1^OR = Odds Ratio, CI = Confidence Interval | | | | | | | | | |
