# Supplementary material for: Association between healthy lifestyle factors and risk of chronic diarrhea: A cross-sectional study using NHANES 2007 to 2010 data
Source: Medicine (Baltimore). 2026 May 29;105(22):e49045. doi: 10.1097/MD.0000000000049045 (PMC13225563; doi:10.1097/MD.0000000000049045)
Supplement: Supplementary file 3 [file medi-105-e49045-s004.docx]

|  | model1 | | | model2 | | | model3 | | |
| --- | --- | --- | --- | --- | --- | --- | --- | --- | --- |
| **Characteristic** | **OR**^1^ | **95% CI**^1^ | **p-value** | **OR**^1^ | **95% CI**^1^ | **p-value** | **OR**^1^ | **95% CI**^1^ | **p-value** |
| **healthy_lifestyle** |  |  |  |  |  |  |  |  |  |
| *4-5* | — | — |  | — | — |  | — | — |  |
| *2-3* | 1.92 | 1.37, 2.70 | **<0.001** | 1.76 | 1.26, 2.46 | **0.002** | 1.72 | 1.16, 2.56 | **0.010** |
| *0-1* | 3.09 | 1.44, 6.61 | **0.005** | 2.74 | 1.32, 5.73 | **0.009** | 2.31 | 1.11, 4.82 | **0.028** |
| ^1^OR = Odds Ratio, CI = Confidence Interval | | | | | | | | | |
